# Use of Botulinum Toxin A in the Treatment of Lower Urinary Tract Disorders: A Review of the Literature

**DOI:** 10.3390/toxins8040088

**Published:** 2016-03-23

**Authors:** David C. Moore, Joshua A. Cohn, Roger R. Dmochowski

**Affiliations:** Department of Urologic Surgery, Vanderbilt University Medical Center, Nashville, TN 37232-2765, USA; joshua.cohn@vanderbilt.edu (J.A.C.); roger.dmochowski@vanderbilt.edu (R.R.D.)

**Keywords:** botulinum toxin, overactive bladder, neurogenic bladder, benign prostatic hyperplasia, detrusor sphincter dyssynergia, interstitial cystitis

## Abstract

Botulinum neurotoxin (BoNT) is used to treat a variety of ailments, and its therapeutic application in lower urinary tract disorders (LUTDs) is well studied. Robust evidence supporting the efficacy and tolerability of BoNT in the treatment of neurogenic detrusor overactivity (NDO) and non-neurogenic overactive bladder (OAB) has led to regulatory approval for these conditions. Use of BoNT in the treatment of interstitial cystitis/bladder pain syndrome, chronic pelvic pain, and detrusor sphincter dyssynergia has demonstrated some promise, but is still evolving and off-label for these indications. Trials to date do not support the use of BoNT for benign prostatic hyperplasia. This comprehensive review outlines the mechanisms of BoNT in the treatment of LUTDs in adults and presents background and updated data examining the efficacy and adverse events associated with the use of BoNT in common urologic applications.

## 1. Introduction

Botulinum neurotoxin (BoNT) is formed by the Gram-positive, anaerobic spore forming bacteria *Clostridium botulinum* and is responsible for human botulism. Clinical applications of BoNT injections include chronic migraines, chronic pain, head and neck dystonias, strabismus, hyperhidrosis, anal fissures, and many others [1]. Use of BoNT in lower urinary tract disorders (LUTDs) was first studied by Dykstra *et al.* in 1988 for the treatment of detrusor external sphincter dyssynergia (DSD) [2]. Following this, investigations into the multiple urologic applications of BoNT amplified. This comprehensive review will focus on the mechanism of action of BoNT, urologic administration, safety, and the data supporting its applications in LUTDs most relevant to practicing urologists.

## 2. Botulinum Toxin and Mechanism of Action

BoNT exerts its activity by prohibiting the release of neurotransmitters from autonomic and somatic nerve endings. BoNT is the most potent known neurotoxin as measured by LD_50_, a product of its unique structure and specificity. If even 1 g of the purified toxin were evenly dispersed and inhaled, it could kill 1 million people [3]. The active 150 kDa polypeptide has three separate domains with distinct functions: (1) the *C*-terminus, which binds pre-synaptic membranes, (2) the *N*-terminus (L-chain), a specific zinc endopeptidase and (3) the middle domain, which facilitates translocation of the L-chain into the cytosol [4]. Translocation of the toxin is correlated with synaptic activity and, thus, the most active nerves are preferentially affected [1].

The seven identified sub-types (A–G) of the L-chain are specific intracellular proteases, with BoNT-A used most commonly for therapeutic applications. BoNT-A cleaves synaptosomal-associated protein (SNAP-25), which is necessary for fusion of synaptic vesicles at the cellular membrane, thus specifically preventing the SNARE-mediated release of neurotransmitters into the synaptic cleft [3]. BoNT-A prevents the release of several neurotransmitters (acetylcholine, adenosine triphosphate (ATP) substance P), as well as downregulates purinergic and capsaicin receptors on afferent neurons, all of which have been implicated in the pathophysiology of NDO and OAB [5,6].

BoNT-A binds with high affinity to synaptic vesicle protein 2 (SV2) and, thus, its activity is limited to cells that express this receptor. Immunofluorescent studies of the healthy human bladder have demonstrated SV2 expression in nearly all cholinergic parasympathetic fibers, half of sensory fibers, and none in urothelial or muscular cells [7]. Likewise, the effect of BoNT-A has been postulated to involve both efferent, parasympathetic pathways and afferent, nociceptive pathways. BoNT does not cause neuronal death, and the effect is temporary as the toxin is inactivated and degraded with time [8]. The commercially available BoNT-A preparations are Botox^®^ (onabotulinumtoxin A, Allergan Pharmaceuticals, Parsippany-Troy Hills, NJ, USA), Dysport^®^ (abobotulinumtoxinA, Ipson Biopharm, Paris, France), and Xeomin^®^ (IncobotulinumtoxinA, Merz Pharmaceuticals, Frankfurt am Main, Germany). While no clinical dose conversion studies exist, it is generally accepted that one unit of onabotulinumtoxin A (onaBoNT-A) is equivalent to 3–5 units of abobotulinumtoxin A (aboBoNT-A) based on comparative effectiveness [9,10,11]. There are too few studies using incobotulinumtoxin A to speculate on dose equivalence.

## 3. Administration and Injection Technique

BoNT-A is administered via intradetrusor injection under local, regional or general anesthesia using a rigid or flexible cystoscope. This procedure can be completed safely in the clinical setting for most patients, although select patients, including those at risk for autonomic dysreflexia, should have closer monitoring in an operative suite. While no protocol regarding the location and number of injections is universally accepted, a technique using a flexible cystoscope with an ultrafine needle has been previously described and adopted by these authors and others [12,13,14]. Ten to thirty minutes prior to injection, 30 mg of 2% lidocaine are instilled into the bladder. For Food and Drug Administration approved indications, 100 U onaBoNT-A diluted in 10 mL preservative-free saline (OAB) or 200 U onaBoNT-A diluted in 20 mL preservative-free saline (NDO) are then injected 1 mL per site separated by a distance of 1 to 1.5 cm. Locations of injections for other indications are described separately.

The increasing evidence of BoNT-A effect on sensory nerves has led to increased focus on trigonal and suburothelial injections. Injections to the trigone have traditionally been spared out of concern for producing vesicoureteral reflux (VUR). Despite this, several studies have shown trigonal injections to be safe and effective without evidence of VUR [15,16]. However, a recent meta-analysis comparing trigonal and extratrigonal injection technique in patients with NDO and idiopathic detrusor overactivity (IDO) found no significant differences with regard to adverse effects or short-term efficacy, suggesting that patient-specific factors and dosing likely matter more for response to BoNT-A than location of injection [17].

As intravesical injection of BoNT-A is invasive and procedurally time-consuming, alternative strategies for topical administration of BoNT-A are being investigated [18]. Given the toxin′s large size, topical application does not permit access to submucosal nerve endings. However, a recent double-blind, placebo-controlled, multicenter trial of liposome-encapsulated onaBoNT-A, an intravesical instillation, has shown statistically significant reductions in OAB symptom scores, micturition frequency, and urgency episodes at four weeks [19]. Such administration may reduce the risk of hematuria, pain, urinary retention, and urinary tract infection (UTI). While the depth of penetration of liposomal-encapsulated onaBoNT-A is yet unknown, this preparation is assumed to reach only the urothelium and suburothelium. Its efficacy supports the theory that BoNT-A does not solely block acetylcholine at parasympathetic nerve endings, and its action is at least partially mediated by urothelial cells or suburothelial afferent nerves [20].

## 4. Safety

Despite the incredible potency of BoNT, the toxin is highly specific for peripheral nerves and does not spread from its site of local injection in significant quantities to cause systemic symptoms [1]. Systemic BoNT toxicity is rare, often associated with higher doses or underlying disease [21]. Signs include impaired vision, extremity weakness, dry mouth, dysphagia, and constipation. Absolute contraindications to BoNT use include active urinary tract infection and hypersensitivity to the toxin or its components. Relative contraindications to BoNT injection include pregnancy, motor neuropathies, and concomitant use of drugs that affect the neuromuscular junction (*i.e.*, aminoglycosides). The most common adverse events (AEs) following BoNT injection in LUTD will be reviewed as relevant to each indication.

## 5. Neurogenic Detrusor Overactivity

Patients with NDO often have severe OAB symptoms with incontinence, and are at risk for elevated detrusor pressures and decreased compliance with associated potential for upper urinary tract deterioration. Ideal candidates for BoNT-A exhibit symptoms of OAB and urge urinary incontinence (UUI), are resistant to or intolerant of anticholinergics, are willing and able to perform clean intermittent catheterization (CIC), and do not have markedly elevated detrusor leak point pressures (DLPPs) or severely decreased detrusor compliance. The most robust data supporting the use of BoNT-A in NDO comes from two double-blind, placebo-controlled, phase-three studies, which were conducted after several phase-two studies demonstrated efficacy in this setting [22,23]. These two pivotal studies by Cruz *et al.* and Ginsberg *et al.* will be discussed together, as their data has previously been pooled for analysis [24,25,26].

A total of 691 patients with either multiple sclerosis (MS) or spinal cord injury (SCI) who had >14 episodes of urinary incontinence (UI) per week were included. Patients must have first experienced intolerance or inefficacy of an appropriately-dosed anticholinergic for >1 month duration. Patients on these medications were permitted to continue them at a constant dose, while those who had discontinued them remained off anticholinergic therapy. Patients were then randomized to receive 30 × 1 mL trigone-sparing injections of onaBoNT-A 200 U, 300 U, or placebo. The primary endpoint was change in UI episodes per week, and secondary outcomes included urodynamics (UDS) findings and Incontinence Quality of Life (I-QOL) score.

There were statistically significant decreases in UI episodes per week in the 200 U (−22.6 MS, −19.6 SCI) and 300 U (−24.0 MS, −18.2 SCI) groups when compared to placebo (−14.0 MS, −6.4 SCI). The percentage of patients who were dry at six weeks also increased with 200 U (41.5% MS, 30.9% SCI) and 300 U (44.2% MS, 35.9% SCI) when compared to placebo (10.7% MS, 7.3% SCI). There was also a reduction in the number of voluntary voids per week in the MS population who were not performing CIC prior to onaBoNT-A. Both MS and SCI patients noted significant increases in their I-QOL scores compared to placebo [26].

Repeat UDS studies were performed six weeks after injection, which are reported here as the range of values taken from both studies. Patients treated with 200 U and 300 U showed increased maximum cystometric capacity (MCC) compared to placebo (151 to 157 mL, 157.2 to 168 mL and 6.5 to 18 mL, respectively). Patients also experienced significant reductions in the number of involuntary detrusor contractions (IDC), and for those with IDO, the maximum detrusor pressure during first IDC (P_detmaxIDC_) was reduced (−28.5 to −35.1 cm/H20, −26.9 to −33.3 cm/H20, −2.4 to 7.9 cm/H20 for 200 U, 300 U and placebo, respectively). Data regarding detrusor compliance were not available. These urodynamic findings were similar among both etiologies, although patients with SCI experienced a greater reduction in P_detmaxIDC_ which is significant when considering long-term risk of upper urinary tract damage.

The most frequent AE was UTI across all patients, which was defined by positive urine culture; the authors did not differentiate asymptomatic bacteriuria from symptomatic infections. In SCI patients, the reported incidence of UTI was similar between onaBoNT-A and placebo (44.8% and 49.5%, respectively). However, among patients with MS there was a significantly increased rate of UTI in the treatment (53.5%) *versus* placebo (29.2%) groups. This difference is possibly secondary to increased rates of urinary retention among treated patients. Most patients with SCI already performed CIC prior to treatment. However, in MS patients, need for CIC after treatment was 40% with 200 U and 51% with 300 U, compared to 17% with placebo. Among those initiating CIC, average frequency was 2.5 times per day, and patient satisfaction was not impacted by need for CIC [26].

To evaluate long-term efficacy of onaBoNT-A injections for NDO, Kennely *et al.* conducted a three-year, prospective, multicenter extension study on 396 of the original study patients [27,28]. The extension study demonstrated sustained improvements in UI episodes per week and I-QOL score. The number of new patients initiating CIC decreased dramatically with each treatment; patients who did not require CIC after three treatments never went on CIC. Reported AEs and AE rates were similar, and no new safety concerns were raised. With 200 U dosing, the median duration of treatment effect was nine months. One small study evaluated 26 patients who had undergone a mean 7.1 BoNT-A injections and found that 26% went on to require definitive surgical treatment for their neurogenic bladder, suggesting that efficacy may decline with repeated injections [29].

Kennely, Cruz and Ginsberg found no statistically significant differences in efficacy between 200 U and 300 U onaBoNT-A, and a placebo-controlled, dose-response study in patients with 50, 100, and 200 U onaBoNT-A noted 200 U to be the most effective dose [30]. This has led to the approval of onaBoNT-A injections for NDO at a starting dose of 200 U by the FDA and similar recommendations by other international consensus agencies [31]. The majority of studies, thus far, have focused on onaBoNT-A; however, limited randomized, placebo-controlled, double-blind studies utilizing aboBoNT-A have demonstrated efficacy with this preparation as well [32,33].

## 6. Non-Neurozgenic Overactive Bladder

Non-neurogenic Overactive Bladder is a symptom complex defined by “urinary urgency, usually accompanied by frequency and nocturia, with or without urgency urinary incontinence, in the absence of UTI or other obvious pathology” [34,35]. While not life-threatening, OAB is a chronic condition and can have a profound impacts on health-related quality of life [36]. First line treatment is behavioral therapy, and second line treatment options include a multitude of anti-muscarinics and β**_3_** adrenoceptor agonists [35]. BoNT-A is considered third line therapy, reserved for patients with moderate to severe OAB symptoms who fail first and second line therapies and are willing to tolerate the risk of having to perform CIC.

Initial efficacy of BoNT-A in the treatment of OAB was demonstrated by several randomized, multi-center, double-blinded trials [37,38,39]. The largest of these, a phase-two dose-ranging study by Dmochowski *et al.* [38], compared 50 U, 100 U, 150 U, 200 U, and 300 U onaBoNT-A to placebo. Doses of 100 U or greater resulted in durable improvements in UUI episodes per week, as well as other OAB symptoms. Doses of 150 U or greater did not result in differences in efficacy relative to 100 U dosing and were associated with dose-dependent increases in post-void residual (PVR) up to 200 U. A PVR of 200 mL or greater seemed to be highly correlated with need for CIC, as well as AEs including UTI and urinary retention.

Phase-two trials demonstrating the efficacy and tolerability of 100 U onaBoNT-A subsequently led to two large, double-blind, placebo-controlled, phase-three studies. Results from these two pivotal studies by Chapple *et al.* and Nitti *et al.* were subsequently pooled for analysis [40,41,42]. A total of 1097 patients with ≥3 UUI episodes per three days and ≥8 micturitions per day were randomized to receive 20 × 0.5 mL trigone sparing injections of onaBoNT-A 100 U or placebo into the detrusor. All patients had failed anticholinergic therapy, with the majority of patients (82.2%) demonstrating inadequate efficacy and a smaller percentage suffering intolerable side effects. OAB symptoms were recorded in patient diaries, and perceptions of treatment benefit were rated on the Treatment Benefit Scale (TBS) at follow-up.

At 12 weeks, the onaBoNT-A arm demonstrated significant improvements in UUI episodes per day relative to placebo (−2.80 *vs.* −0.95; *p* < 0.001). Additionally, 60.5% of patients experienced >50% improvement in UUI, and 27.1% achieved 100% improvement (continence). Positive response on the TBS was significantly higher after treatment when compared to placebo (61.8% *vs.* 28.0%; *p* < 0.001). Patients on active treatment also experienced significant improvements relative to placebo in daily episodes of urgency and frequency. On sub-group analysis, these effects persisted despite the number of prior anticholinergics used or the reason for discontinuation of medical therapy [42]. The median duration of effect among treated patients was 24 weeks.

The most common AE was UTI (25.5% onaBoNT-A *vs.* 9.6% placebo), defined by a positive urine culture (10^5^ cfu/mL), regardless of symptoms. Other significant AEs were dysuria (10.9% onaBoNT-A *vs.* 7.0% placebo), bacteruria (8.0% *vs.* 3.5%) and urinary retention (5.8% *vs.* 0.4%), which was defined as a PVR ≥200 mL. Patients were started on CIC for PVR ≥ 200 mL with symptoms or PVR > 350 mL regardless of symptoms.. Overall, rates of CIC were low (6.5% onaBoNT-A *vs.* 0.4% placebo), with similar numbers requiring CIC for ≤6 weeks and >12 weeks (2.5% and 2.7%, respectively).

A separate dose ranging, randomized controlled trial (RCT) by Rovner *et al.* obtained baseline UDS in all patients followed by repeat measurements at 12 and 36 weeks post treatment [43]. 76.0% of enrolled patients had demonstrable detrusor overactivity (DO) on baseline UDS. Relative to placebo, patients treated with 100 U onaBoNT-A showed increased MCC at 12 weeks (+71.0 mL *vs.* 49.5 mL). Significant increases in detrusor compliance at MCC or prior to terminal IDC were noted at week 12 (+63.0 *vs.* −22.8 mL/cm H_2_O). No difference in efficacy was noted between patients who had demonstrated DO on UDS *vs.* those who did not. In patients with urodynamic DO, there were similar reductions in proportion with demonstrable DO at week 12 between patients on treatment *vs.* placebo (34.9% *vs.* 31.4%). Similar results were observed with regard to detrusor pressure at first IDC. Thus, while some UDS parameters are improved following onaBoNT-A treatment for OAB, the clinical utility of these findings is unclear. Consequently, routine UDS testing in patients with OAB is not required prior to treatment with BoNT-A.

To determine the difference in efficacy between therapies, Visco *et al.* conducted a double-blind, double-placebo-controlled RCT comparing anticholinergic therapy (AT) to BoNT-A injections [44]. 249 women with an average 5.0 UUI episodes/3 day were randomized to receive oral AT + intravesical placebo injections or 100 U onaBoNT-A + oral placebo. Episodes of UUI improved in both AT and onaBoNT-A groups (3.4 *vs.* 3.3 per day, respectively) as did QOL. More patients treated with BoNT-A experienced resolution of UUI (27% *vs.* 13%, *p* = 0.003). At six months, the majority of patients in both arms had adequate control of symptoms (71% AT *vs.* 70% onaBoNT-A). When comparing adverse events, AT patients were more likely to experience dry mouth (46% *vs.* 31%), but less likely to experience UTI (13% *vs.* 33%) and require CIC (0% *vs.* 5%). Thus, 100 U onaBoNT-A has comparable efficacy to AT in the treatment of OAB, albeit with higher rates of complete continence.

To evaluate long-term efficacy of onaBoNT-A injections for OAB, Mohee *et al.* conducted a seven-year retrospective evaluation of 137 patients treated with onaBoNT-A at a single center [45]. Patients were initially treated with 200 U, which was subsequently lowered as guidelines emerged. The mean re-treatment interval was eight months, but the majority of patients (61.3%) discontinued treatment at 36 months. The principal reason for discontinuing treatment were intolerance of side effects (need for CIC, urinary retention, UTIs). Younger patients and those with persistent incontinence were more likely to discontinue treatment. In small retrospective series, Smits *et al.* evaluated patients who had failed BoNT-A treatment for OAB referred for sacral neuromodulation (SNM) and noted treatment efficacy of SNM in 70% (14/20) of patients, suggesting a role for SNM in patients who are unable or do not wish to continue therapy with BoNT-A [46].

These data demonstrate conclusive efficacy for onaBoNT-A as a treatment option for refractory OAB. While the longevity of treatment response may be limited, it does not appear to compromise success of neuromodulation. While most studies, thus far, have focused on onaBoNT-A, one small study does demonstrate efficacy of aboBoNT-A [47]. Taken together, these findings have led to FDA approval of onaBoNT-A injections for patients with OAB who have failed second line therapy at a starting dose of 100 U, with similar recommendations by other international consensus agencies [31,35].

## 7. Benign Prostatic Hyperplasia

Use of BoNT-A for benign prostatic hyperplasia (BPH) was first investigated by Maria *et al.* in 2003 [48]. This placebo-controlled RCT compared 30 patients receiving 200 U onaBoNT-A or saline via perineal intraprostatic injection. The authors noted a statistically significant 65% reduction in American Urological Association symptom score (AUA-SS) and 51% reduction in PSA values in the treatment group. Several other small studies reported improvements in symptom scores and flow rates [49,50,51]. Crawford *et al.* subsequently conducted a double-blinded RCT comparing 100 U to 300 U onaBoNT-A injected transrectally under ultrasound guidance [52]. None of the 134 patients randomized were on medical therapy at time of treatment; both doses demonstrated significant improvements in AUA-SS and flow rates.

As a follow-up to these initial investigations, Marberger *et al.* and McVary *et al.* conducted two large randomized, placebo-controlled trials on 492 total patients with peak flow rates <15 mL/s and International Prostate Symptom Score (IPSS) >12 [53,54]. Marberger compared 100 U, 200 U, and 300 U onaBoNT-A via perineal or transrectal administration, whereas McVary administered 200 U under transrectal guidance. The procedure was well tolerated, with the most common AEs being hematuria and hematospermia.

Both studies reported significant improvement in symptom scores and flow rates from baseline after injection; however, there were no differences between the treatment and placebo arms. A recent meta-analysis of the three placebo controlled trials also corroborated this profound placebo effect, asserting no benefit for onaBoNT-A in the treatment of BPH [55]. Thus, studies to date do not support the use of onaBoNT-A as first-line treatment for BPH, and it is not included in the most updated AUA guidelines [56]. Nevertheless, small separate sub-analyses did demonstrate improvement in flow rates in patients on concurrent medical therapy (McVary), and improvements in symptom scores in patients who had been treated previously with alpha-blockers (Marberger). Consequently, there may yet be a subset of patients on maximal medical management and poor candidates for surgical intervention who may benefit from intraprostatic onaBoNT-A injection. An ongoing RCT comparing onaBoNT-A to optimized medical therapy (the PROTOX study) may provide additional details [57].

## 8. Interstitial Cystitis/Bladder Pain Syndrome

The Interstitial Cystitis/Bladder Pain Syndrome (IC/BPS) complex is defined as “an unpleasant sensation (pain, pressure, discomfort) perceived to be related to the urinary bladder, associated with lower urinary tract symptoms of more than six weeks duration, in the absence of infection or other identifiable causes” [58]. No standardized treatment regimen exists, as patient response is highly variable to a multitude of therapies, including behavioral modification, physical therapy, pharmacologic agents, and surgical treatments such as cystoscopy with hydrodistention (HD) or fulguration of Hunner′s lesions, sacral neuromodulation and cystectomy. BoNT-A injections were first used after several observational studies reported efficacy in this patient population [59,60,61]. Several follow-up prospective studies reported efficacy of onaBoNT-A, often in conjunction with hydrodistention, for the treatment of IC/BPS [62,63,64,65]. As these studies varied in their injection locations and inclusion criteria, consensus opinion remained elusive.

Two RCTs were subsequently conducted to further delineate which patients might benefit from BoNT-A. The first by Kuo and Chancellor compared onaBoNT-A injections to the posterior and lateral bladder walls + HD two weeks later at doses of 100 U and 200 U to HD alone [66]. Enrolled patients (*N* = 67) had failed conventional treatments and remained on pentosan sulfate throughout the trial. Success of onaBoNT-A + HD based on global response assessment (GRA) at three months was 80% in the 200 U arm and 72% in the 100 U arm, compared to 48% for HD alone. Only patients treated with onaBoNT-A demonstrated improvements in bladder pain visual analog scale (VAS) and MCC. These effects diminished by 12 and 24 months. Importantly, rates of AEs (dysuria, elevated PVRs) were higher in the 200 U group, despite no significant difference in efficacy.

Kuo *et al.* followed this study with a double­blind, placebo­controlled, multicenter trial randomizing 60 patients to 20 × 0.5 mL suburothelial injections to the posterior and lateral bladder wall of either 100 U onaBoNT-A or saline immediately followed by HD [67]. All patients had failed six months of conventional therapy, and ulcerative forms of IC/PBS were excluded. At 8 weeks, the onaBoNT-A group demonstrated a significant reduction in VAS compared to placebo (−2.6 *vs.* −0.9; *p* = 0.02). MCC was also increased in onaBoNT-A treated patients (+67.8 *vs.* −45.4; *p* = 0.020), however, other UDS metrics as well as GRA and subjective symptoms of frequency/nocturia did not differ between treatment arms. The most common AEs were dysuria (40%), UTI (5%) and urinary retention (2.5%). This AE profile, including the uncommon need for CIC, is corroborated by other studies [62,64,68]. While larger placebo-controlled trials will be required for FDA approval, these early trials suggest potential efficacy of 100 U onaBoNT-A for the treatment of IC/BPS. Subsequently, the AUA recommends onaBoNT-A at a starting dose of 100 U as a fourth-line treatment option for IC/BPS in patients willing to CIC and refractory to other treatment modalities [69]. As IC/BPS is widely varied in its presentation and symptomatology, it would follow that certain patients would be more likely to respond to BoNT-A therapy. This should be a focus of future studies.

## 9. Chronic Pelvic Pain

Chronic pelvic pain (CPP) in men and women is defined by chronic or persistent pain for at least six months duration perceived to originate in the organs of the pelvis [70]. Chronic prostatitis/chronic pelvic pain syndrome (CP/CPPS) and IC/BPS all fall within the spectrum of CPP, each defined by pain referred specifically to the respective organs. Treatment is varied and includes psychological counseling, multimodal pharmacologic analgesia, biofeedback and pelvic floor physiotherapy, among others. The pathophysiology of CPP is unclear but likely involves pelvic floor muscle (PFM) tenderness and spasm, as well as chronic central nervous system hypersensitivity and neurogenic inflammation [71,72,73]. Consequently, BoNT-A has been investigated as a treatment modality given its unique ability to target these pathways.

Efficacy of BoNT-A in the treatment of CP/CPPS was first investigated in small studies reporting relief of prostate pain and urethral hyperalgesia after transurethral perisphincteric injection of 200 U onaBoNT-A [74]. Later, a small placebo controlled RCT showed modest improvements in pain scores after injection of 100 U onaBoNT-A into the perineal body and bulbospongiosus muscle [75]. Subsequently, a double-blind, placebo-controlled RCT was conducted to evaluate BoNT use in men with CP/CPS refractory to 4–6 weeks of conventional medical therapy [76]. Enrolled patients (*N* = 60) were randomized to receive transurethral intraprostatic injection of 100 U onaBoNT-A or saline. Treated patients demonstrated significant improvements in National Institutes of Health Chronic Prostatitis Symptom Index (NIH-CPSI) total and subscale scores, AUA-SS, QOL scores, urinary frequency and nocturia compared to placebo. Most notably at six months, marked improvement in VAS scores (82.2% lower *vs.* 2.4% higher) and NIH-CPSI pain scores (79.97% lower *vs.* 4.7% higher) were seen in patients receiving onaBoNT-A compared to placebo, respectively. No major AEs were observed, with transient hematuria noted in only two patients. Another small uncontrolled randomized trial recently showed improved efficacy with transrectal ultrasound guided BoNT-A injection compared to transurethral injection [77]. A phase-two study of 40 men with CP/CPS is currently underway comparing 300 U onaBoNT-A to 1% lidocaine (ClinicalTrials.gov identifier: NCT00529386).

BoNT-A use in the treatment of CPP in women, specifically PFM spasm, has received limited study. Jarvis *et al.* first reported efficacy of 40 U onaBoNT-A injected into the puborectalis and pubococcygeus muscles under conscious sedation in 12 women with CPP [78]. Subsequently, Abott *et al.* conducted a double-blind, placebo-controlled RCT in women with CPP >2 years duration and evidence of pelvic muscle spasm [79]. Patients (*N* = 60) were randomized to receive 80 U onaBoNT-A (20 U/mL) or saline in four 1 mL injections to PFMs as described previously. Treated patients showed improvements in dyspareunia and nonmenstrual pelvic pain, whereas the placebo arm showed only improvement in dyspareunia. Patients treated with onaBoNT-A demonstrated a larger decrease in pelvic floor pressure compared to placebo. Two patients developed incontinence after treatment, and no AEs were observed in the placebo arm.

More recently, an open-label, prospective pilot study investigated electromyography (EMG) guided onaBoNT-A injections in 21 women with CPP [80]. Using this technique, a needle attached to an EMG electrode is used to localize spastic PFMs and target these sites for injection, potentially using up to 300 U onaBoNT-A in total. The majority of patients noted improvement in QOL scores, and 83.3% reported improvement in dyspareunia at 24 weeks. Resting vaginal pressures also decreased, and less point tenderness was noted on physical exam of the PFMs. Significant AEs were constipation (28.6%), UI (4.8%) and fecal incontinence (4.8%). A systemic review of these studies and others also reported benefit of onaBoNT-A in patients with CPP related to PFM spasm [81].

While small in number, these preliminary studies suggest efficacy of BoNT-A injections for the treatment of CPP. As this is a heterogenous condition, it is not surprising that reported injection locations, methods and dosages are highly variable. Larger RCTs are needed to standardize treatments for these various indications. Further research will elucidate which pathophysiologic parameters are being affected by BoNT-A injection and identify subsets of patients most likely to benefit from therapy.

## 10. Detrusor Sphincter Dyssynergia

Detrusor Sphincter Dyssynergia (DSD) refers to spastic and uncoordinated contraction of the external sphincter upon voiding seen in many patients with SCI and MS. Dysfunctional voiding can lead to incomplete emptying, thus increasing the risks of UTI and upper urinary tract damage. While often managed with CIC and less commonly sphincterotomy, BoNT-A injection into the external sphincter was one of the earliest urologic indications evaluated [2,82,83]. Subsequently, multiple small observational studies demonstrated efficacy of 100 U onaBoNT-A for treatment of DSD when injected via transurethral, transrectal or transperineal routes [84,85,86,87]. An small RCT (*N* = 13) comparing 100 U onaBoNT-A to lidocaine in SCI patients with DSD showed improvements in PVR at 30 days after treatment (−159.4 mL after onaBoNT-A *vs.* −49.8 mL after lidocaine), although mean post-treatment PVR remained elevated at 105 mL [88]. The authors noted improvements in mean urethral pressure (MUP) and other objective measurements of DSD. A recent meta-analysis of these studies in patients with SCI noted a mean PVR decrease from 251.8 to 153.0 mL after treatment with BoNT-A lasting up to six months, as well as a reduction in UTIs and CIC in some series [89]. AEs described in these studies have been minimal.

Similarly, Gallien *et al.* conducted a multi-center, double-blind RCT comparing 100 U onaBoNT-A to placebo via single transperineal injection in patients with MS [90]. Enrolled patients (*N* = 86) had MS with DSD and elevated PVRs (100–500 mL). At 30 days, there was no difference in PVR between treatment and placebo, but those treated with onaBoNT-A did demonstrate increased voiding volume (+54%, *p* = 0.02), pre-micturition P_det_ (229%, *p* = 0.02) and maximal P_det_ (221%, *p* = 0.02). Several heterogenous small studies have demonstrated efficacy of onaBoNT-A injections in reducing PVR in patients with SCI and DSD, an effect that was not observed in patients with MS. This may be explained by differences in study design, pathophysiology of these disease states, and/or the sex distribution of patients with these conditions (*i.e.*, SCI patients are majority male, MS majority female). Although quality data are lacking, onaBoNT-A injections to the external sphincter appear to be a reasonable short-term treatment option for patients with DSD [91]. Further research is needed before more definitive recommendations can be made.

## 11. Conclusions

Evidence for the use of BoNT-A, specifically onaBoNT-A, in the treatment of LUTDs continues to accumulate. Multiple robust, well-designed trials have demonstrated clear efficacy of BoNT-A in the treatment of NDO and OAB, subsequently leading to FDA approval of onaBoNT-A for these conditions and widespread clinical use. Studies to date do not support the use of BoNT-A in the treatment of BPH. Although evidence is limited, existing data suggest BoNT-A may be a promising option for some patients with CPP and IC/BPS. Similarly, small studies demonstrate safety and efficacy of BoNT-A use in the treatment of DSD, but certainly larger, well-designed RCTs are needed. As this field rapidly evolves, future research will focus on efficacy in non-approved indications, alternative delivery systems, and elucidating the histophysiologic changes in LUTDs in relation to the BoNT-A mechanism of action.

## Abbreviations

The following abbreviations are used in this manuscript:

aboBoNT-A: abobotulinumtoxin A
AE: adverse event
AT: anticholinergic therapy
AUA-SS: American Urologic Association symptom scores
BoNT: Botulinum neurotoxin
BPH: benign prostatic hyperplasia
CIC: clean intermittent catheterization
CP/CPPS: chronic prostatitis/chronic pelvic pain syndrome
CPP: chronic pelvic pain
DLPPs: Detrusor leak point pressures
DO: detrusor overactivity
DSD: Detrusor Sphincter Dyssynergia
EMG: electromyography
GRA: global response assessment
HD: hydrodistention
I-QOL: Incontinence Quality of Life
IC/BPS: Interstitial Cystitis/Bladder Pain Syndrome
IDC: involuntary detrusor contractions
IDO: idiopathic detrusor overactivity
IPSS: International Prostate Symptom Score
LUTDs: lower urinary tract disorders
MCC: maximum cystometric capacity
MS: multiple sclerosis
MUP: mean urethral pressure
NDO: neuropathic detrusor overactivity
NIH-CPSI: National Institutes of Health Chronic Prostatitis Symptom Index
onaBoNT-A: onabotulinumtoxin A
P_detmaxIDC_: maximum detrusor pressure during first IDC
PFM: pelvic floor muscle
PVR: post void residual
RCT: randomized controlled trial
SCI: spinal cord injury
SNM: sacral neuromodulation
SV2: synaptic vesicle protein 2
TBS: Treatment Benefit Scale
UDS: urodynamics
UI: urinary incontinence
UTI: urinary tract infection
UUI: urge urinary incontinence
VAS: visual analog scale
VUR: vesicoureteral reflux
